# Green Synthesis for Carbon Quantum Dots via *Opuntia ficus-indica* and *Agave maximiliana*: Surface-Enhanced Raman Scattering Sensing Applications

**DOI:** 10.1021/acsomega.3c02735

**Published:** 2023-09-07

**Authors:** A. Navarro-Badilla, G. Calderon-Ayala, Y. Delgado-Beleño, M. C. Heras-Sánchez, R. Britto Hurtado, J. E. Leal-Pérez, A. Hurtado-Macias, M. Cortez-Valadez

**Affiliations:** † Departamento de Física, Universidad de Sonora, Rosales y Blvd. Luis Encinas S/N, Hermosillo, Sonora 83000, Mexico; ‡ Universidad Estatal de Sonora, Rosales No. 189 Col. Centro, Hermosillo 83100, Mexico; § Grupo de Espectroscopía Óptica y Láser, Universidad Popular del Cesar, Valledupar, Cesar 200001, Colombia; ∥ Departamento de Matemáticas, Universidad de Sonora, Rosales y Blvd. Luis Encinas S/N, Hermosillo, Sonora 83000, Mexico; ⊥ CONAHCYT-Departamento de Investigación en Física, Universidad de Sonora, Apdo. Postal 5-88, Hermosillo, Sonora 83190, Mexico; # Universidad Autónoma de Sinaloa, Fuente de Poseidón y Prol. Ángel Flores S/N., Los Mochis, Sinaloa 81223, Mexico; ¶ Centro de Investigación en Materiales Avanzados S.C. (CIMAV), Miguel de Cervantes 120, Chihuahua, Chihuahua 31136, Mexico

## Abstract

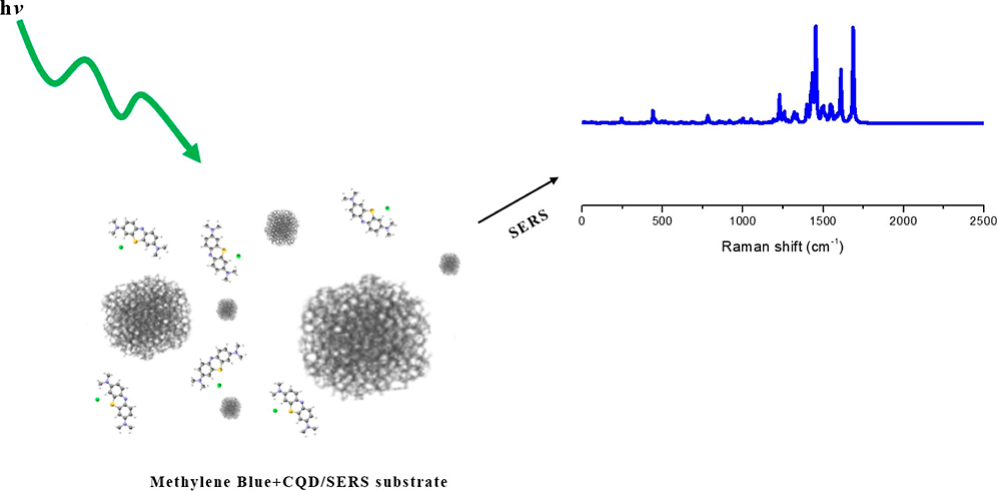

In this study, we present an alternative method for synthesizing carbon quantum dots (CQDs) using a green synthesis approach via extracts from *Agave maximiliana* and *Opuntia ficus-indica(Ofi)*. The extracts from both plants were used as the carbon source for the CQDs. The synthesis method employs mesoporous zeolite 4A as a refractory for the thermal treatment of the samples. Transmission electron microscopy analysis established that the size of the CQDs shows a narrow distribution centered around 2 nm with a maximum size of less than 3 nm for both cases. The CQDs exhibit absorption bands associated with π–π* transitions located around 220 nm. In both cases, photoluminescence (PL) phenomenon was detected by irradiating the samples with a UV wavelength and detecting emissions close to the blue wavelength. Additionally, both kinds of CQDs were tested as surface-enhanced Raman scattering (SERS) substrates against methylene blue (MB), indicating an enhancement associated with ring deformation and stretching modes of the v(C–C) and v(C–N) bonds located around 1400 and 1620 cm^–1^, respectively. Complementarily, in the framework of density functional theory, H_2*n*_C_2(2*m*+1)_ structures (with *n* = 3–5 and *m* = 1–3) were used as a theoretical representation of CQDs in interaction with the MB molecule. It is used for developing the analysis of charge transfer effects between both systems and for specifying elements that generate the SERS effect associated with the chemical enhancement mechanism.

## Introduction

Carbon quantum dots (CQDs) are low-dimensional systems characterized by their stability and functionalization capacity for developing potential applications such as biosensing, drug delivery, nanomedicine, biomarkers, photocatalysis, and others.^1–4^ Currently, different synthesis methods for obtaining and stabilization include microwave processes, thermal decomposition, hydrothermal treatments, laser ablation, and ultrasonic treatments.^5–10^ After the publication of Zhu and colleagues, where soy milk was considered to obtain CQDs, a significant number of results have emerged regarding green synthesis as an effective, low-cost, and competitive method for producing this kind of low-dimensional systems.^11^ Through green synthesis, renewable natural resources have been used to obtain CQDs, such as lemon peel, watermelon, *Aloe vera*, *onion*, *Ginkgo biloba*, *Mangifera indica*, and others.^12–17^ Implementing these synthesis route guarantees the substitution of regularly toxic or highly toxic compounds with components from plants, fruits, vegetables, roots, leaves, and stems, which regularly help biocompatibility.^18^ Therefore, a simple method could be obtained using organic sources in the synthesis process, representing an environmental-friendly protocol with the possibility of large-scale preparation. Even expensive reagents and sophisticated equipment are not required to generate low-dimensional systems.

The size of the CQDs plays an important role in developing their different applications. It is necessary to state that zeolite is an additional component not regularly used in hydrothermal methods. It can generate an effect on confinement in obtaining low-dimensional systems. Also, zeolite is considered a mesoporous material, and its nanosized channels act as a molecular sieve.^18^ Under specific experimental conditions, this can influence the narrow distribution of the final sizes of CQDs within the framework of the hydrothermal synthesis methodology.

On the other hand, carbon nanostructures have been used to develop potential applications in surface-enhanced Raman scattering (SERS) sensing of pyridine, biological systems, cell lines, glucose, among others.^19–21^ Recently, Lei et al. used carbon nanostructures for SERS sensing of methylene blue (MB).^22^ They found a predominant intensification in the vibrational mode associated with the stretching vibration of the C–C ring of MB.

Nevertheless, an important tool for studying the charge transfer (CT) mechanism has been the density functional theory (DFT).^23^ This theory allows for determining minimum-energy electronic configurations between interacting systems and predicting energy levels and geometries of frontier orbitals and absorption bands in the UV–vis spectrum associated with CT.^24,25^ Recent DFT results by Kong et al. indicated that after an interaction between small carbon clusters and the pyridine molecule, CT between both systems occurs because the electron density is transferred from the clusters to the pyridine.^26^ Due to this behavior, the SERS effect occurs within the framework of the chemical enhancement mechanism (CEM).

In this work, we used a green route via *Opuntia ficus-indica* (*Ofi*) and *Agave maximiliana* (*Agave*) to obtain CQDs with a narrow size distribution. The particles were experimentally evaluated as SERS substrates on MB. Moreover, theoretical elements are presented within the framework of DFT, showing evidence of CT between clusters of carbon ring C_*n*(Ring)_ and the MB molecule.

## Materials and Methods

### Synthesis of Carbon Quantum Dots

The individual infusions of *Agave* and *Ofi* were obtained by singly mixing 20 g of pulp from each cactus with distilled water. Each infusion was exposed to magnetic stirring at 70 °C for 1 h. It is to prevent temperatures above boiling and to avoid modifying the components of each plant extract. The solution containing plant components was obtained by filtration. Subsequently, this solution is mixed with zeolite 4A using magnetic stirring (155 °C). In this way, the air is released from the zeolite channels, and the solution can easily flow through the channels and promote the sieving behavior. In order to optimize the synthesis process and energy consumption, the sample is subjected to thermal treatment at 200 °C for 2 h. After the carbonization process, the resulting solid is pulverized using an agate mortar. The powdered sample is mixed with 30 mL of deionized water and centrifuged at 11,000 rpm. The resulting solution was filtered, and only the liquid from the top of the solution was collected to avoid precipitates in the sample.

The optical absorption analysis was performed using a VELAB 5100 UV spectrometer in the 200–800 nm range. The LABram HR Evolution Raman spectrometer from Horiba with λ = 780 nm was used for the SERS effect analysis. The commercial MB was selected as a probe molecule.

The TEM JEOL JEM2010F equipment was used for the analysis of the morphology and structural parameters of the CQDs.

### Theoretical Methodology

Complementarily, a theoretical analysis is considered for the energy levels between the MB molecule and carbon structures representing the surface of CQDs. We determined some molecular descriptors showing electronic behavior and its relationship with the SERS effect. For this, the C_6_H_6_, C_10_H_8_, and C_14_H_10_ structures interacted with the MB molecule (C_16_H_18_ClN_3_S) within the framework of DFT and under the B3LYP approximation level (Becke 3-parameter Lee–Yang–Parr) in combination with the 6-31G basis set. In all cases, the minimum local energy of the system was found, and the vibrational spectrum was evaluated to ensure the presence of only positive frequencies. The interacting systems were perturbed with an incident wavelength of 785 nm. Moreover, to analyze and locate the contribution of CT between both systems, time dependence-self consistent field (TD-SCF) was considered to solve molecular excited states (*N* = 20).

## Results and Discussion

**Figure 1:**
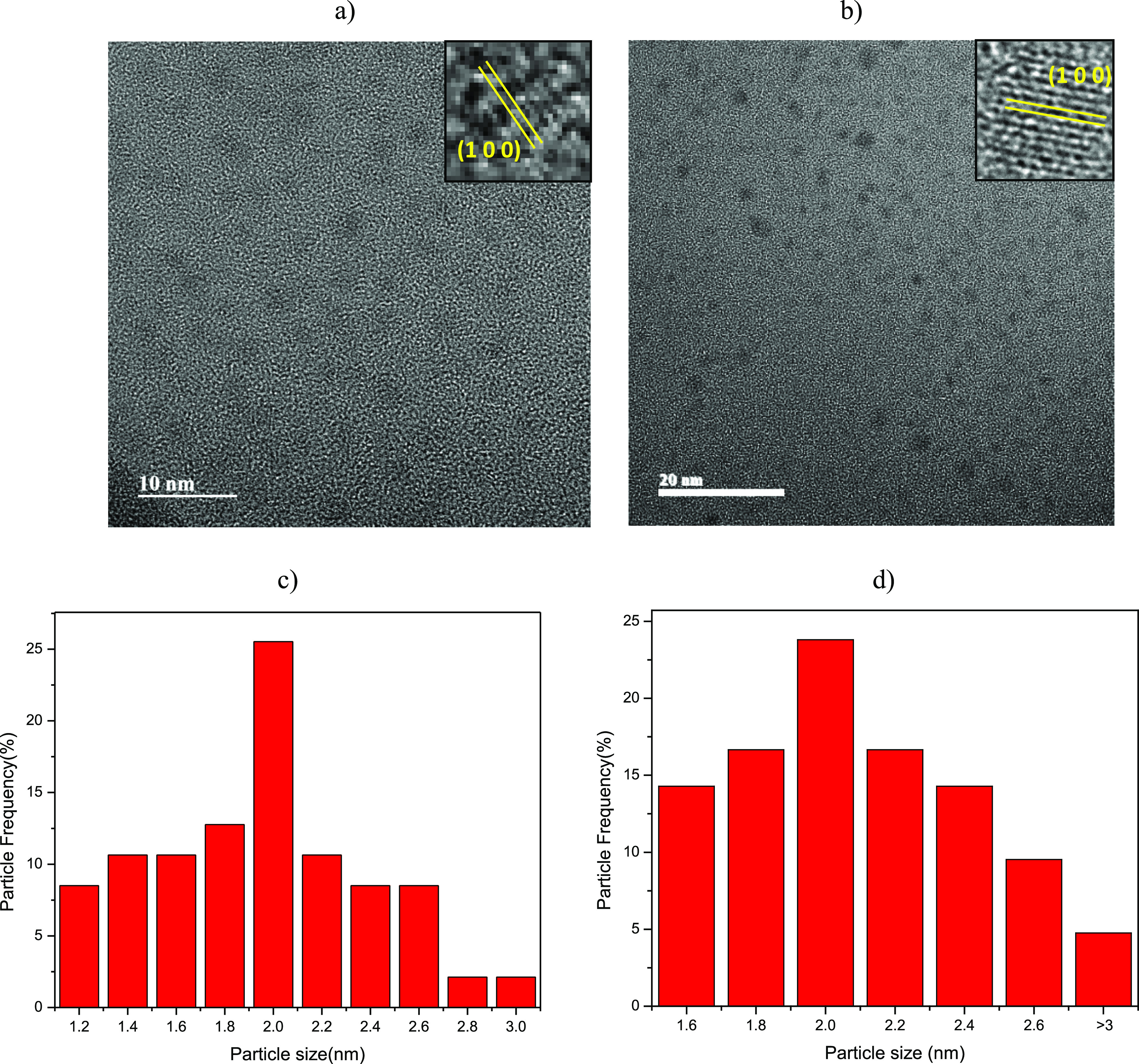
TEM images of CQDs obtained through (a) *O. ficus-indica* and (b) *A. maximiliana*. Particle size histogram: (c) corresponding to Figure 1a and (d) corresponding to Figure 1b.

The CQDs showed predominant sizes above 2 nm for both the *Ofi* and *Agave* extracts, as shown in Figure 1. No aggregation is appreciated in both cases, and the particles are observed with a regular distribution. However, when *Ofi* is used, a narrow predominance in particle size is obtained. It is well known that temperature plays a significant role in this synthesis process. Nevertheless, the proposed synthesis method used the same thermal treatment conditions. Therefore, we assume that the higher content and proportion of carbon source components in the *Ofi* extract such as citric acid, carbohydrates, ascorbic acid, flavonoids, and others may influence the particle size effects.^27^

The study of the chemical composition in *Agave* varieties is very limited. However, the high fiber content and many glycosides have been found in some varieties.^28^ In other studies, the presence of glycosides has been one of the main factors causing the formation of CQDs, achieving particles with good distribution, stability, and isolation.^29^

Only particles below or around 3 nm were observed in the TEM analysis. It may be associated with the zeolite 4A matrix and the structural characteristics of its channels, acting as sieves and limiting the early stages of CQD growth. The magnitudes in the interplanar distances allowed the identification of the Miller indices (100) in *Ofi* and *Agave*, corresponding to the values of 2.14 and 2.15 Å of graphite 2H, respectively.^30^

CQDs are known to exhibit absorption bands located approximately between 220 and 338 nm in the UV–vis spectrum.^31,32^ These are associated with sp^2^ hybridization with π–π* transitions from the C=C bond. Furthermore, *n*–π* transitions have been seen around 300 nm, which can be extended to regions of about 400 nm. Some authors have indicated that the functionalization of CQDs, collateral effects of the synthesis methods, and precursors used in obtaining these particles influence shifts in absorption bands toward lower and higher energy levels. Such is the case when synthesizing CQDs through glucose and exposure to microwaves, obtaining absorption bands at 285 nm.^33^ On the other hand, some precursor carbon sources may influence the absorption bands to shift toward higher energies located at 242 nm associated with π–π* transitions in CQDs.^34^

**Figure 2:**
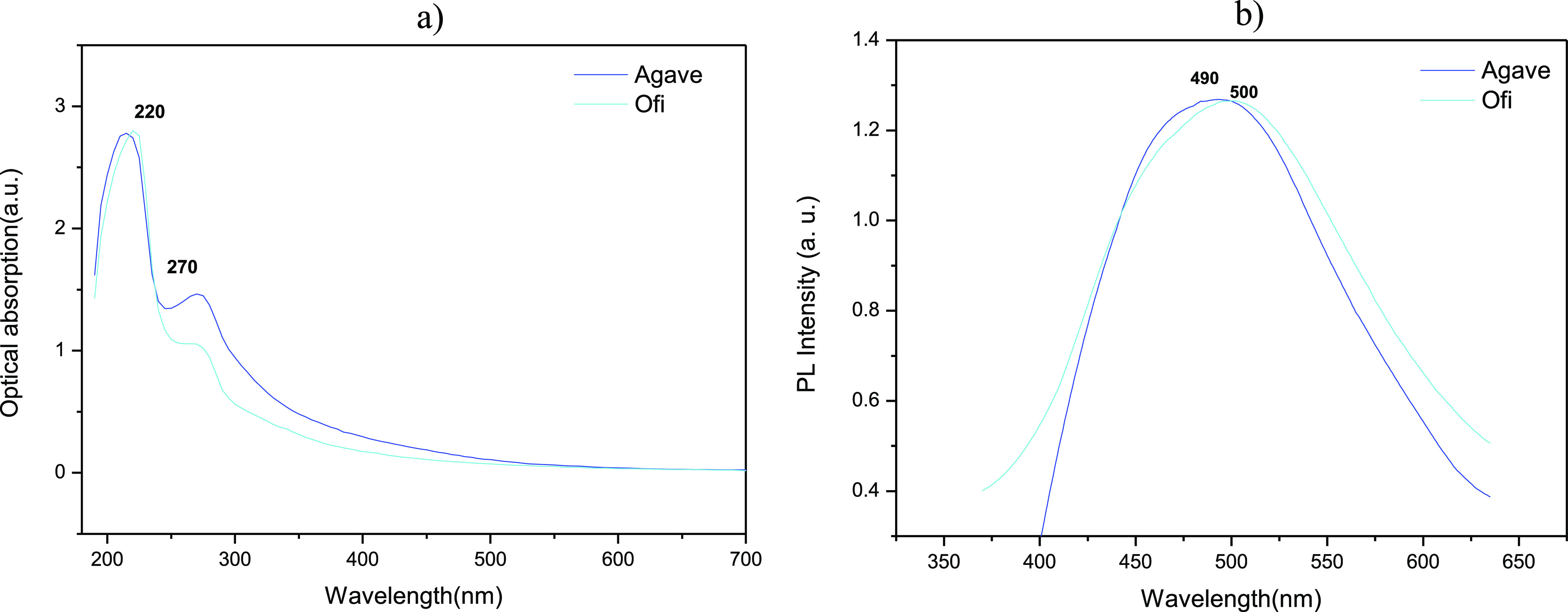
(a) Optical absorption of QDs of *Agave* and *Ofi*, blue and light blue, respectively. (b) Photoluminescence spectra of both species under excitation wavelengths of 380 and 350 nm for *Agave* and *Ofi*, respectively.

When the UV–vis spectrum of CQDs in *Agave* and *Ofi* was studied, a well-defined band located at approximately 220 nm was obtained, associated with π–π* transitions, as shown in Figure 2a. In this regard, a slight presence of this kind of transition was observed when using the *Ofi* extract. The band detected at 270 nm disappears for oven heat treatments higher than 400 °C. This band could indicate the presence of residual components of the extract that remained uncarbonized at lower temperatures. Furthermore, the CQDs in solution were subjected to incident radiation with wavelengths centered at 380 and 350 nm (*Agave* and *Ofi*, respectively). Confirming the photoluminescent properties, Figure 2b shows the emission spectra centered at 490 and 500 nm, associated with electronic transitions of emitting elements in the CQDs in *Agave* (blue light) and *Ofi* (blue), respectively.

**Figure 3:**
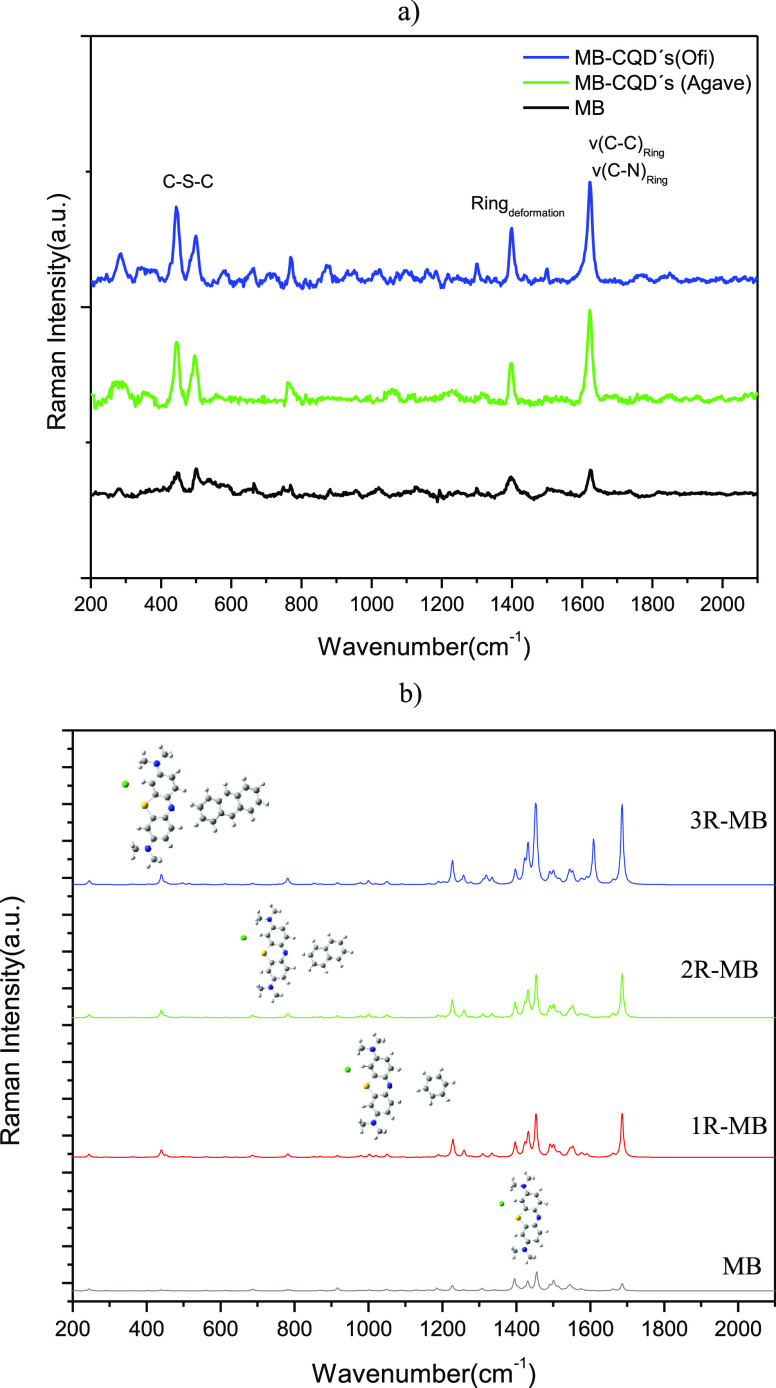
Raman spectrum of MB-CQDs: (a) experimental and (b) predicted by DFT.

Complementarily, the CQDs were evaluated against MB as the SERS substrates. Figure 3a shows the experimental Raman spectrum of the MB (black line). In this spectrum, two twin bands are observed at around 500 cm^–1^. These are associated with the C–N–C bending mode and may be susceptible to SERS effect factors.^35^ A band near 1400 cm^–1^ has been associated with in-plane ring deformation.^35^ The characteristic band of MB is located at 1625 cm^–1^, which is in the v(C–C) and v(C–N) stretching mode regions of the ring. This vibrational mode exhibits high susceptibility to the SERS effect, as observed in Figure 3. The Raman spectra of CQDs derived from the *Agave* extract and MB are shown in Figure 3 (green line). It showed a lower relative intensity of the band centered at 1625 cm^–1^ of MB. For CQDs obtained from the *Ofi* extract (blue line), the relative intensity of this band was slightly higher. In both cases, an intensification of the twin bands at around 500 cm^–1^ is observed.

In addition, the SERS spectra of the MB-CQDs interaction were predicted. Layered structures of *n* carbon rings were evaluated, considering cases with *n* = 1–3 (C_*n*(Ring)_) to represent the CQDs. In relation to this, the local minimum energy configurations were obtained in the interaction of the MB-CQDs systems under the B3LYP approximation level in combination with the 6-31G basis set. The predicted Raman spectra are shown in Figure 3b. The characteristic modes of the MB susceptible to intensification upon interaction with the CQDs are compared with the vibrational modes obtained from the C_*n*(Ring)_–MB interaction representation. The results are given in Table 1.

**Table 1:** Theoretical/Experimental Comparative Raman Modes

	experimental Raman modes (cm^–1^)			predicted Raman modes(cm^–1^)				refs
vibrational mode	MB-CQDs (Agave)	MB*	MB-CQDs (*Ofi)*	MB	MB–C_1(Ring)_	MB–C_2(Ring)_	MB–C_3(Ring)_	
C–S–C_symm1_	443	450	443	439.06	439.64	439.71	439.95	(36)
C–S–C_symm2_	497	500	500	495.97	497.09	496.01	496.53	(36)
Ring_deformation_	1400	1398	1400	1395.26	1397.20	1397.12	1397.62	(35)
ν(C–C)Ringν(C–N)Ring	1620	1626	1623	1686.61	1686.41	1686.32	1686.06	(37)

The molecular orbitals HOMO and LUMO of the considered systems are shown in Table 2. On the left side, we can observe the energy levels of the HOMO and LUMO orbitals of the individual systems. On the right side, we observe a modification in the energy levels following the interaction with the MB molecule. This provides possibilities for CT between both systems to occur. A more detailed analysis is presented when the electronic density is distributed over the active regions of the system.

**Table 2:** Energy of HOMO/LUMO before and after the MB Interaction

	before MB interaction, eV		after MB interaction, eV	
structure	HOMO	LUMO	HOMO	LUMO
C_1(Ring)_	–6.754	0.102	–4.340	–3.386
C_2(Ring)_	–5.825	–0.962	–4.344	–3.388
C_3(Ring)_	–5.255	–1.634	–4.343	–3.392
MB	–4.301	–3.343		

Moreover, the molecular orbitals that are susceptible to electron transfer are listed in Table 3. In this case, the electronic transitions of each interacting system C_*n*(ring)_–MB are analyzed. For the cases of C_1_–MB and C_2_–MB, the carbon ring transitions toward MB were located between approximately 415 and 430 nm. For the case of C_3_–MB, these transitions were located between 378 and 436 nm. For this system, electronic transitions of MB toward C_3_ were predicted between 512 and 522 nm. In all analyzed cases, it was determined that HOMO is one of the main orbitals for donating electrons from MB to C_*n*(ring)_. Likewise, the LUMO electronically distributed over the MB presents a greater susceptibility to accept electrons from C_*n*(ring)_.

**Table 3:**
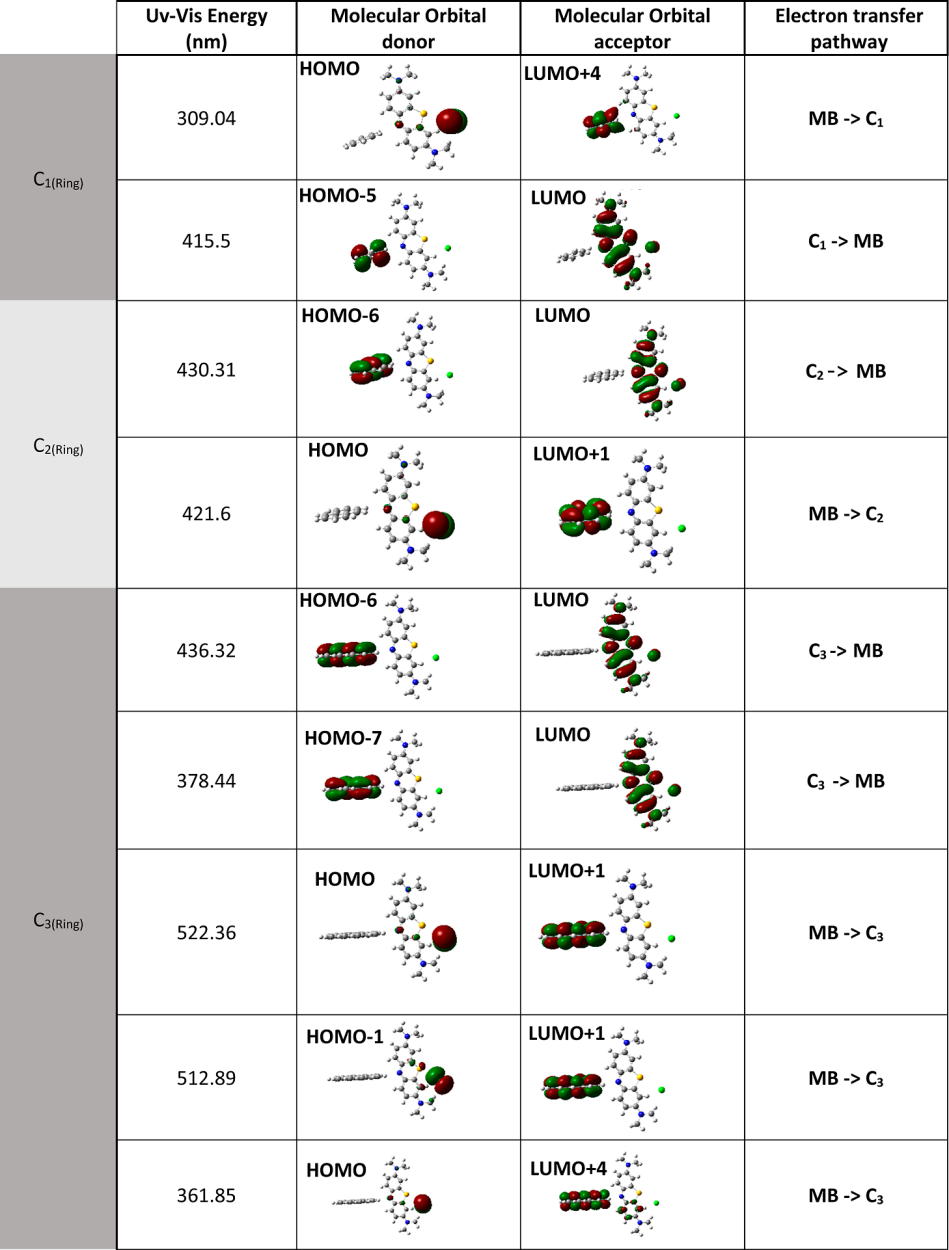
Electron Transfer Pathway after the MB Interaction

## Conclusions

The proposed method ensured the synthesis of CQDs through a green synthesis using extracts from *Ofi* and *Agave* with narrow sizes centered at 2 nm. The effect of the mesoporous zeolite matrix as a refractory and sieve is believed to have impacted the size confinement of the CQDs. The presence of absorption bands associated with π–π* and *n*–π* transitions and emission bands in the PL spectrum reaffirmed the stable presence of these kinds of particles in the colloidal solution.

As indicated in the Raman spectra, the experimental evaluation of CQDs as SERS substrates on MB resulted in the intensification of four vibrational modes. These were associated with symmetric C–S–C stretching modes, carbon ring deformations, and stretching modes (C–C and C–N) in the carbon ring. DFT modeling of C_*n*(ring)_ as a CQDs allowed for quantifying the energy levels in the frontier orbitals before and after interaction with the MB molecule (approximately −4.3 and −3.3 eV for HOMO and LUMO, respectively). The prediction of the location of the vibrational modes susceptible to SERS intensification was also made. CT manifestations were predicted in UV–vis regions. Electronic transitions from the carbon ring to the MB molecule (for cases C_1_ and C_2_) were found around 415–430 nm. Additionally, similar transitions were located in the region between 378 and 436 nm for the case of C_3_. For the C_3_–MB system, electronic transitions from the MB to the C_3_ were predicted between 512 and 522 nm. Therefore, the SERS effect in this kind of system can be related to the chemical enhanced mechanism (CEM).

These results suggest that CQDs can be used as a colloidal solution for SERS sensing to analyze MB-derived molecules with expected intensification regions in the mid-infrared.

## Data Availability

The experimental and theoretical data supporting this study are available on request from corresponding author M. Cortez-Valadez.
